# Higher Oxygenation Is Associated with Improved Survival in Severe Traumatic Brain Injury but Not Traumatic Shock

**DOI:** 10.1089/neur.2022.0065

**Published:** 2023-01-23

**Authors:** Daniel P. Davis, Barbara McKnight, Eric Meier, Ian R. Drennan, Craig Newgard, Henry E. Wang, Eileen Bulger, Martin Schreiber, Michael Austin, Christian Vaillancourt

**Affiliations:** ^1^ Logan Health EMS, Kalispell, Montana, USA.; ^2^ Department of Emergency Medicine, UC San Diego Medical Center, San Diego, California, USA.; ^3^ Department of Biostatistics, University of Washington, Seattle, Washington, USA.; ^4^ Rescu, Li Ka Shing Knowledge Institute, St. Michael's Hospital, Toronto, Ontario, Canada.; ^5^ Institute of Medical Science, Faculty of Medicine, University of Toronto, Toronto, Ontario, Canada.; ^6^ Center for Policy and Research in Emergency Medicine, Department of Emergency Medicine, Oregon Health & Science University, Portland, Oregon, USA.; ^7^ Department of Emergency Medicine, University of Alabama at Birmingham, Birmingham, Alabama, USA.; ^8^ Department of Surgery, University of Washington, Seattle, Washington, USA.; ^9^ Department of Surgery, Oregon Health & Science University, Portland, Oregon, USA.; ^10^ Ottawa Hospital Research Institute, University of Ottawa, Ottawa, Ontario, Canada.

**Keywords:** emergency medical services, hyperoxemia, hypoxemia, shock, traumatic brain injury

## Abstract

Pre-hospital resuscitation of critically injured patients traditionally includes supplemental oxygen therapy to address potential hypoxemia. The objective of this study was to explore the association between pre-hospital hypoxemia, hyperoxemia, and mortality in patients with traumatic brain injury (TBI) and traumatic shock. We hypothesized that both hypoxemia and hyperoxemia would be associated with increased mortality. We used the Resuscitation Outcomes Consortium Prospective Observational Prehospital and Hospital Registry for Trauma (ROC PROPHET) database of critically injured patients to identify a severe TBI cohort (pre-hospital Glasgow Coma Scale [GCS] 3–8) and a traumatic shock cohort (systolic blood pressure ≤90 mm Hg and pre-hospital GCS >8). Arterial blood gas (ABG) obtained within 30 min of hospital arrival was required for inclusion. Patients with hypoxemia (PaO_2_ <80 mm Hg) and hyperoxemia (PaO_2_ >400 mm Hg) were compared to those with normoxemia (PaO_2_ 80–400 mm Hg) with regard to the primary outcome measure of in-hospital mortality in both the TBI and traumatic shock cohorts. Multiple logistic regression was used to calculate odds ratios (ORs) after adjustment for multiple covariables. In addition, regression spline curves were generated to estimate the risk of death as a continuous function of PaO_2_ levels. A total of 1248 TBI patients were included, of whom 396 (32%) died before hospital discharge. Associations between hypoxemia and increased mortality (OR, 1.8; 95% confidence interval [CI], 1.2–2.8; *p* = 0.008) and between hyperoxemia and decreased mortality (OR, 0.6; 95% CI, 0.4–0.9; *p* = 0.018) were observed. A total of 582 traumatic shock patients were included, of whom 52 (9%) died before hospital discharge. No statistically significant associations were observed between in-hospital mortality and either hypoxemia (OR, 1.0; 95% CI, 0.4–2.4; *p* = 0.987) or hyperoxemia (OR, 1.9; 95% CI, 0.6–5.7; *p* = 0.269). Among patients with severe TBI but not traumatic shock, hypoxemia was associated with an increase of in-hospital mortality and hyperoxemia was associated with a decrease of in-hospital mortality.

## Introduction

Traumatic injuries account for tremendous morbidity and mortality in North America, representing the leading cause of death among young adults and the most important contributor to years of productive life lost across all age groups.^1–3^ Traumatic brain injury (TBI) and traumatic shock account for the vast majority of deaths, particularly in the initial 24 h post-injury, and thus represent primary targets for early resuscitative efforts.^3–5^ This includes aggressive airway and ventilatory therapies as well as hemorrhage control and administration of fluids and blood products to reverse potential oxygenation and perfusion deficits.^6,7^

Whereas these therapies have been considered the standard of care for several decades, it is notable that definitive evidence to support aggressive resuscitation is lacking. In fact, rapid reversal of traumatic hypotension with intravenous fluids may be associated with increased mortality by dislodging clots, diluting clotting factors, and lowering temperature.^8–10^ In addition, multiple studies suggest an association between early intubation and increased mortality among TBI patients.^11–13^ Although it is possible that intubation is merely a marker for more severe injuries, several physiological explanations exist to explain the association with poor outcomes. The high prevalence of hyperventilation after intubation is well documented and appears to result in cerebral vasoconstriction and ischemia.^14,15^ An additional hypothesis concerns the potential for excessive oxygen to result in free radical formation as part of the reperfusion injury cascade. Hyperoxemia has been associated with adverse outcomes in other critically ill patients, including birth asphyxia, traumatic shock, post-surgical patients, and cardiopulmonary arrest.^16–20^ However, previous investigations exploring an association between hyperoxemia and outcomes among patients with traumatic injuries have been inconsistent.^19–24^

The objectives of this study were to examine the association between both hypoxemia and hyperoxemia and in-hospital mortality from severe TBI and traumatic shock using a prospective registry database as part of the Resuscitation Outcomes Consortium (ROC). We hypothesized that normal oxygen levels would be associated with better survival than either hypoxemia or hyperoxemia in both TBI and traumatic shock.

## Methods

### Design

This was a retrospective cohort analysis using prospectively collected data as part of the PROPHET (ROC Prospective Observational Prehospital and Hospital Registry for Trauma) study. Waiver of informed consent was granted from the sites' institutional review boards.

### Setting

Details regarding the ROC study group have been presented elsewhere.^25^ In brief, ROC represents an 11-site (eight United States, three Canada) study collaborative exploring optimal therapies in cardiac arrest and severe traumatic injury. Whereas the primary purpose of ROC has been to perform interventional trials, prospective observational data were collected to explore hypotheses that might help guide the selection and design of interventional trials and support data collection infrastructure within each site.^26^ The PROPHET database represents a cohort of high-acuity trauma patients, with detailed pre-hospital and in-hospital data collection from 114 emergency medical services (EMS) agencies transporting to 56 trauma hospitals across North America.^25^ The defined objectives of PROPHET included to: 1) maintain a comprehensive ongoing data infrastructure to facilitate the design, implementation, and interpretation of ROC trauma trials; 2) evaluate the ability of pre-hospital factors to predict in-hospital measures of injury severity in trauma patients with life-threatening injury; and 3) evaluate the relationships between pre-hospital injury characteristics, patient characteristics, EMS and regional structure, processes of care, and outcome in trauma patients with life-threatening injury. Patients were enrolled in the PROPHET from January 1, 2010 through June 30, 2011.

### Subjects

All subjects from the eight U.S. sites and three Canadian sites entered into the ROC PROPHET database were considered for inclusion. For this analysis, included subjects had either: 1) Glasgow Coma Scale (GCS) score of 3–8 (TBI cohort) or 2) systolic blood pressure (SBP) ≤90 mm Hg at any time during the pre-hospital course. Patients with both SBP < = 90 mm Hg and GCS 3–8 were included in the TBI cohort. In addition, included subjects were required to have vital signs recorded at admission, data from an arterial blood gas (ABG) obtained within 30 min of hospital arrival, and a documented Injury Severity Score (ISS).

### Statistical analysis

This study intended to explore the relationship between extremes of oxygenation and outcome from severe TBI and traumatic shock. We hypothesized that both early hypoxemia and hyperoxemia would be associated with an increase in death before hospital discharge. *A priori* definitions were used for both hypoxemia (PaO_2_ <80 mm Hg) and hyperoxemia (PaO_2_ >400 mm Hg) based on previous investigations.^21,24,27^ Patients with TBI were analyzed separately from patients with traumatic shock. Patients meeting criteria for both TBI and traumatic shock were analyzed as part of the TBI group. For PaO_2_ and PaCO_2_, the first ABG obtained within 30 min of hospital arrival was used for analysis.

Data regarding demographics, mechanism of injury, physiological status, advanced airway insertion, and death before hospital discharge were presented descriptively using counts and percentages for categorical variables and means and standard deviations for most continuous variables. Median and interquartile range were used for SBP because of the issue of “non-detectable” values. The primary hypothesis was explored using multiple logistic regression adjusting for the following covariables: age; sex; site; mechanism of injury (blunt, penetrating); lowest pre-hospital SBP; GCS [TBI cohort], Abbreviated Injury Score (AIS) values for head, chest, and abdomen; ISS; use of an advanced airway (endotracheal tube or supraglottic airway) in the pre-hospital environment; and the presence of either hypocapnia (PaCO_2_ <30 mm Hg) or hypercapnia (PaCO_2_ >50 mm Hg). In-hospital mortality was used as the primary outcome measure for both the TBI and traumatic shock analyses.

Natural cubic splines with four interior knots at the 20th, 40th, 60th, and 80th percentiles were computed and plotted to the relationship between initial PaO_2_ value and death before hospital discharge.^29^ Splines were adjusted for the same covariables as the logistic regression and were plotted along with 95% point-wise confidence intervals (CIs) for values of the covariables listed in the figure legends. The R statistical program (version 3.3.2) was used for all statistical calculations.^30^ Statistical significance was assumed for *p* < 0.05.

## Results

**FIG. 1.**
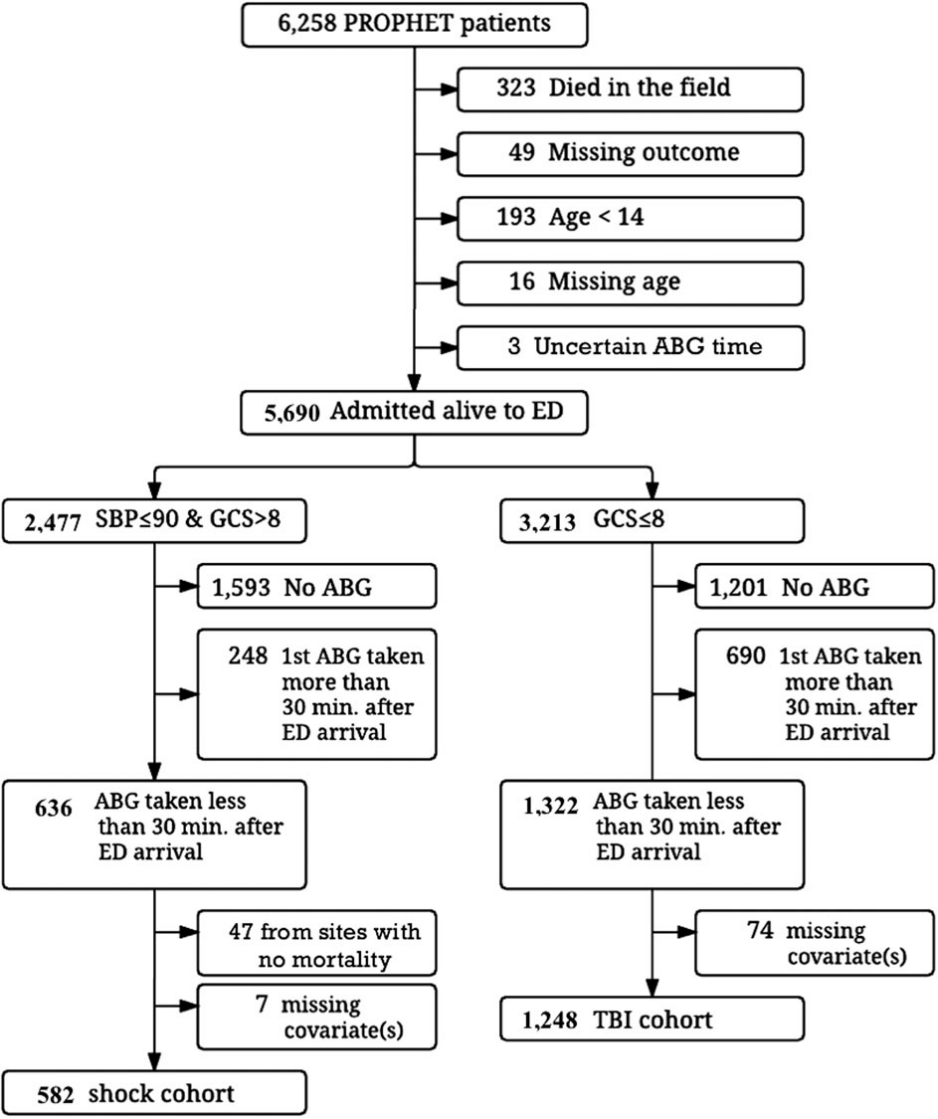
Flow diagram for subject inclusion in this study. ABG, arterial blood gas; ED, emergency department; GCS, Glasgow Coma Scale; SBP, systolic blood pressure; TBI, traumatic brain injury.

A total of 1322 TBI patients (GCS 3–8) with ABG data obtained within 30 min of hospital arrival were identified from the PROPHET database (Fig. 1). A total of 74 patients were excluded because of missing adjustment variables (including 43 without ISS and 28 without SBP), resulting in a final study TBI cohort of 1248 patients. This included 182 patients (14.6%) with hypoxemia (PaO_2_ <80 mm Hg), 819 patients (65.6%) with normoxemia (PaO_2_ 80–400 mm Hg), and 247 patients (19.8%) with hyperoxemia (PaO_2_ >400 mm Hg). Baseline demographic and clinical data are displayed in Tables 1A and 2A. A total of 396 TBI patients (32%) died before hospital discharge.

**Table 1A.** Baseline Demographic and Clinical Data for TBI Patients (GCS <8) with PaO_2_ Obtained ≤30 min of ED Arrival (*n* = 1322), PaO_2_ Obtained >30 min of ED Arrival (*n* = 686), or No PaO_2_ Obtained (*n* = 1201)

	PaO_2_ taken ≤30 minutes after ED arrival (*n = *1322)	PaO_2_ taken >30 minutes after ED arrival (*n = *686)	No PaO_2_ taken (*n = *1201)
*Demographics and injury characteristics*			
Age, y, mean (SD)	41 (19)	41 (19)	42 (20)
Male, *n* (%)	980 (74)	524 (76)	884 (74)
Penetrating injury, *n* (%)	169 (13)	83 (12)	259 (22)
ISS, mean (SD)	26 (16)	25 (15)	17 (17)^**^
> 15, *n* (%)	938 (73)	513 (77)	499 (49)^**^
Head AIS ≥3, *n* (%)	826 (64)	451 (67)	354 (33)^*^
Chest AIS ≥3, *n* (%)	493 (38)	255 (37)	304 (28)^*^
Abdomen AIS ≥3, *n* (%)	171 (13)	73 (11)	76 (7)^*^
*Pre-hospital vital signs and airway*			
Lowest SBP, mmHg, median (IQR)	110 (83, 132)	113 (88, 136)	96 (0, 126)^*^
≤ 90, *n* (%)	416 (32)	190 (29)	552 (49)^*^
First GCS, mean (SD)	5.0 (2.5)	5.2 (2.5)	5.1 (3.0)
Intubated, *n* (%)	564 (43)	219 (32)	314 (26)
Supraglottal, *n* (%)	81(6)	49 (7)	146 (12)
BVM only, *n* (%)	208 (16)	166 (24)	233 (19)
*ED admission vital signs*			
SBP, mmHg, median (IQR)	131 (108, 153)	131 (106, 151)	121 (0, 141)^**^
≤ 90, *n* (%)	192 (15)	100 (15)	350 (34)^**^
GCS, mean (SD)	5.5 (3.8)^*^	5.2 (3.2)^*^	7.4 (5.0)^**^
≤ 0 8, *n* (%)	1001 (81)^*^	517 (84)^*^	598 (61)^**^
*Other ED/hospital measures*			
Intubated in field or ED, *n* (%)	1169 (88)	631 (92)	607 (51)
Hemoglobin, g/dL, mean (SD)	12.5 (2.5)	13.0 (2.4)	NR
INR, mean (SD)	1.36 (1.03)^*^	1.32 (1.08)^*^	NR
Base deficit/excess, mmol/L, median (IQR)	-4.9 (-9.0, -1.9)	-4.5 (-8.0, -2.0)	NR
≤ -6, *n* (%)	540 (41)	259 (39)	NR
Bleed on initial head CT, *n* (%)	709 (59)^*^	408 (65)^*^	NR
Any blood products in first 24h, *n* (%)	483 (37)	255 (37)	179 (15)
Ventilation category			
Hypocapnia (<30 mmHg), *n* (%)	81(6)	35 (5)	NA
Eucapnia (30-50 mmHg), *n* (%)	938 (71)	517 (75)	NA
Hypercapnia (>400 mmHg), *n* (%)	300 (23)	133 (19)	NA
Oxygenation category			
Hypoexemia (<80 mmHg), *n* (%)	204 (15)	74 (11)	NA
Normoxemia (80-400 mmHg), *n* (%)	860 (65)	504 (73)	NA
Hyperoxemia (>400 mmHg), *n* (%)	258 (20)	108 (16)	NA
*Outcomes*			
24-hour mortality, *n* (%)	249 (19)	126 (18)	546 (45)
Discharge GOS ≤3, *n* (%)	865 (65)	479 (70)	710 (59)
In-hospital mortality, *n* (%)	439 (33)	240 (35)	593 (49)

^*^ Missing 5-10%.

NR - Not reported due to >25% missing.

^**^ Missing 10-20%.

NA - No ABG taken.

TBI, traumatic brain injury; GCS, Glasgow Coma Scale; ED, emergency department; SD, standard deviation; ISS, Injury Severity Score; AIS, Abbreviated Injury Scale; SBP, systolic blood pressure; IQR, interquartile range; BVM, bag valve mask; INR, international normalized ratio; CT, computed tomography; GOS, Glasgow Outcome Scale.

**Table 1B.** Baseline Demographic and Clinical Data for Traumatic Shock Patients (SBP <90 mm Hg, GCS >8) with PaO_2_ Obtained ≤30 min of ED Arrival (*n* = 636), PaO_2_ Obtained >30 min of ED Arrival (*n* = 248), or No PaO_2_ Obtained (*n* = 1593)

	PaO_2_ taken ≤30 minutes after ED arrival (*n = *636)	PaO_2_ taken >30 minutes after ED arrival (*n = *248)	No PaO_2_ taken (*n = *1593)
*Demographics and injury characteristics*			
Age, y, mean (SD)	41 (18)	40 (18)	47 (22)
Male, *n* (%)	472 (74)	192 (77)	929 (58)
Penetrating injury, *n* (%)	258 (41)	87 (35)	304 (19)
ISS, mean (SD)	17 (13)	20 (14)	7 (8)
> 15, *n* (%)	296 (47)	143 (59)	202 (13)
Head AIS ≥3, *n* (%)	70 (11)	29 (12)	68 (4)
Chest AIS ≥3, *n* (%)	237 (37)	107 (43)	191 (12)
Abdomen AIS ≥3, *n* (%)	150 (24)	79 (32)	68 (4)
*Pre-hospital vital signs and airway*			
Lowest SBP, mmHg, median (IQR)	80 (70, 88)	80 (69, 88)	84 (78, 90)
First GCS, mean (SD)	14.0 (1.7)^*^	13.9 (1.6)	14.6 (1.0)
Intubated, *n* (%)	62 (10)	4 (2)	15 (1)
Supraglottal, *n* (%)	2 (0)	1 (0)	5 (0)
BVM only, *n* (%)	35 (6)	35 (14)	100 (6)
*ED admission vital signs*			
SBP, mmHg, median (IQR)	113 (90, 134)	102 (85, 125)	114 (99, 130)
≤ 90, *n* (%)	162 (26)	78 (32)	240 (16)
GCS, mean (SD)	13.2 (3.7)^*^	13.3 (3.2)^**^	14.5 (1.8)^***^
≤ 8, *n* (%)	69 (12)^*^	20 (9)^**^	31 (3)^***^
*Other ED/hospital measures*			
Intubated in field or ED, *n* (%)	226 (36)	109 (44)	71 (4)
Hemoglobin, g/dL, mean (SD)	11.8 (2.6)	12.2 (2.3)	NR
INR, mean (SD)	1.26 (0.51)^*^	1.27 (0.57)^**^	NR
Base deficit/excess, mmol/L, median (IQR)	-4.0 (-7.4,-1.6)	-6.0 (-9.0, -3.0)	NR
≤ -6, *n* (%)	217 (35)	130 (53)	NR
Any blood products in first 24h, *n* (%)	285 (45)	140 (56)	150 (9)
Ventilation category			
Hypocapnia (<30 mmHg), *n* (%)	101 (16)	12 (5)	NA
Eucapnia (30-50 mmHg), *n* (%)	485 (76)	201 (81)	NA
Hypercapnia (>400 mmHg), *n* (%)	48 (8)	34 (14)	NA
Oxygenation category			
Hypoexemia (<80 mmHg), *n* (%)	112 (18)	35 (14)	NA
Normoxemia (80-400 mmHg), *n* (%)	465 (73)	176 (71)	NA
Hyperoxemia (>400 mmHg), *n* (%)	59 (9)	37 (15)	NA
*Outcomes*			
24-hour mortality, *n* (%)	25 (4)	14 (6)	32 (2)
In-hospital mortality, *n* (%)	54 (8)	21 (8)	59 (4)

^*^ Missing 5-10%.

NR - Not reported due to >40% missing.

^**^ Missing 10-20%.

NA - No ABG taken.

^***^ Missing 20-25%.

GCS, Glasgow Coma Scale; ED, emergency department; SD, standard deviation; ISS, Injury Severity Score; AIS, Abbreviated Injury Scale; SBP, systolic blood pressure; IQR, interquartile range; BVM, bag valve mask; INR, international normalized ratio.

**Table 2A.** Baseline Demographic and Clinical Data for TBI Patients (GCS <8) with Hypoxemia (PaO_2_ <80 mm Hg), Normoxemia (PaO_2_ 80–400 mm Hg), and Hyperoxemia (PaO_2_ >400 mm Hg) and an ABG Obtained within 30 min of ED Arrival

	Hypoxemia (*n = *204)	Normoxemia (*n = *860)	Hyperoxemia (*n = *258)
*Demographics and injury characteristics*			
Age, y, mean (SD)	38 (17)	42 (19)	38 (19)
Male, *n* (%)	154 (75)	646 (75)	180 (70)
Penetrating injury, *n* (%)	29 (14)	111 (13)	29 (11)
ISS, mean (SD)	30 (17)^*^	25 (17)	24 (15)
> 15, *n* (%)	153 (82)^*^	604 (72)	181 (71)
Head AIS ≥3, *n* (%)	129 (68)^*^	532 (63)	165 (64)
Chest AIS ≥3, *n* (%)	102 (51)	318 (37)	73 (29)
Abdomen AIS ≥3, *n* (%)	35 (18)	109 (13)	27 (10)
*Pre-hospital vital signs and airway*			
Lowest SBP, mmHg, median (IQR)	105 (75, 131)	110 (82, 132)	110 (90, 130)
≤ 90, *n* (%)	77 (38)	274 (33)	65 (26)
First GCS, mean (SD)	4.5 (2.3)	5.1 (2.5)	5.4 (2.6)
Intubated, *n* (%)	85 (42)	350 (41)	129 (50)
Supraglottal, *n* (%)	25 (12)	46 (5)	10 (4)
BVM only, *n* (%)	42 (21)	129 (15)	37 (14)
*ED admission vital signs*			
SBP, mmHg, median (IQR)	122 (92, 152)	132 (108, 154)	134 (116, 152)
≤ 90, *n* (%)	48 (24)	114 (14)	30 (12)
GCS, mean (SD)	4.7 (3.4)^*^	5.8 (4.0)^*^	5.1 (3.2)^*^
≤ 8, *n* (%)	165 (87)^*^	626 (78)^*^	210 (87)^*^
*Other ED/hospital measures*			
Intubated in field or ED, *n* (%)	184 (90)	736 (86)	249 (97)
Hemoglobin, g/dL, mean (SD)	11.7 (2.9)	12.6 (2.4)	12.7 (2.3)
INR, mean (SD)	1.71 (1.59)^*^	1.30 (0.85)	1.30 (0.99)
Base deficit/excess, mmol/L, median (IQR)	-6.0 (-11.3, -2.7)	-5.0 (-9.0, -2.0)	-3.8 (-7.2, -1.2)
≤ -6, *n* (%)	104 (52)	356 (42)	80 (31)
Bleed on initial head CT, *n* (%)	103 (65)^***^	464 (59)^*^	142 (58)
Any blood products in first 24h, *n* (%)	95 (47)	304 (35)	84 (33)
Ventilation category			
Hypocapnia (<30 mmHg), *n* (%)	4 (2)	53 (6)	24 (9)
Eucapnia (30-50 mmHg), *n* (%)	85 (42)	641 (75)	212 (82)
Hypercapnia (>400 mmHg), *n* (%)	114 (56)	164 (19)	22 (9)
*Outcomes*			
24-hour mortality, *n* (%)	79 (39)	142 (17)	28 (11)
Discharge GOS ≤3, *n* (%)	162 (79)	537 (63)	166 (64)
In-hospital mortality, *n* (%)	106 (52)	275 (32)	58 (22)

^*^ Missing 5-10%.

^**^ Missing 10-20%.

^***^ Missing 20-25%.

TBI, traumatic brain injury; GCS, Glasgow Coma Scale; ABG, arterial blood gas; ED, emergency department; SD, standard deviation; ISS, Injury Severity Score; AIS, Abbreviated Injury Scale; SBP, systolic blood pressure; IQR, interquartile range; BVM, bag valve mask; INR, international normalized ratio; CT, computed tomography; GOS, Glasgow Outcome Scale.

**Table 2B.** Baseline Demographic and Clinical Data for Traumatic Shock Patients (SBP <90 mm Hg, GCS >8) with Hypoxemia (PaO_2_ <80 mm Hg), Normoxemia (PaO_2_ 80–400 mm Hg), and Hyperoxemia (PaO_2_ >400 mm Hg) and an ABG Obtained within 30 min of ED Arrival

	Hypoxemia (*n = *112)	Normoxemia (*n = *465)	Hyperoxemia (*n = *59)
*Demographics and injury characteristics*			
Age, y, mean (SD)	43 (18)	40 (18)	36 (16)
Male, *n* (%)	82 (73)	340 (73)	50 (85)
Penetrating injury, *n* (%)	32 (29)	191 (41)	35 (59)
ISS, mean (SD)	18 (13)	16 (13)	20 (14)
> 15, *n* (%)	59 (53)	204 (44)	33 (57)
Head AIS ≥3, *n* (%)	19 (17)	44 (9)	7 (12)
Chest AIS ≥3, *n* (%)	48 (43)	170 (37)	19 (33)
Abdomen AIS ≥3, *n* (%)	29 (26)	99 (21)	22 (38)
*Pre-hospital vital signs and airway*			
Lowest SBP, mmHg, median (IQR)	80 (70, 88)	80 (70, 88)	80 (60, 84)
First GCS, mean (SD)	14.0 (1.7)^*^	14.1 (1.6)^*^	13.3 (2.1)^**^
Intubated, *n* (%)	10 (9)	38 (8)	14 (24)
Supraglottal, *n* (%)	0 (0)	2 (0)	0 (0)
BVM only, *n* (%)	9 (8)	23 (5)	3 (5)
*ED admission vital signs*			
SBP, mmHg, median (IQR)	108 (85, 126)	115 (91, 135)	118 (92, 138)
≤ 90, *n* (%)	35 (32)	114 (25)	13 (23)
GCS, mean (SD)	13.3 (3.8)^*^	13.6 (3.3)^*^	10.4 (5.2)^**^
≤ 8, *n* (%)	14 (13)^*^	38 (9)^*^	17 (33)^**^
*Other ED/hospital measures*			
Intubated in field or ED, *n* (%)	42 (38)	140 (30)	44 (75)
Hemoglobin, g/dL, mean (SD)	11.9 (2.1)	11.9 (2.6)	11.0 (2.6)
INR, mean (SD)	1.23 (0.35)^**^	1.25 (0.54)^*^	1.36 (0.49)^*^
Base deficit/excess, mmol/L, median (IQR)	-3.9 (-6.8, -0.5)	-4.0 (-7.2, -1.7)	-5.8 (-10.7, -2.5)
≤ -6, *n* (%)	32 (29)	156 (34)	29 (50)
Any blood product in first 24h, *n* (%)	50 (45)	196 (42)	39 (66)
Ventilation category			
Hypocapnia (<30 mmHg), *n* (%)	7 (6)	86 (19)	8 (14)
Eucapnia (30-50 mmHg), *n* (%)	87 (78)	350 (75)	48 (81)
Hypercapnia (>400 mmHg), *n* (%)	17 (15)	28 (6)	3 (5)
*Outcomes*			
24-hour mortality, *n* (%)	7 (6)	14 (3)	4 (7)
In-hospital mortality, *n* (%)	12 (11)	34 (7)	8 (14)

^*^ Missing 5-10%.

^**^ Missing 10-20%.

SBP, systolic blood pressure; GCS, Glasgow Coma Scale; ABG, arterial blood gas; ED, emergency department; SD, standard deviation; ISS, Injury Severity Score; AIS, Abbreviated Injury Scale; IQR, interquartile range; BVM, bag valve mask; INR, international normalized ratio.

A total of 636 traumatic shock patients (SBP <90 mm Hg at any time during pre-hospital course and GCS >8) with ABG data obtained within 30 min of hospital arrival were identified from the PROPHET database (Fig. 1). A total of 7 patients were excluded because of missing adjustment variables. In addition, 47 subjects from three sites (Ottawa, Pittsburgh, and Portland) were excluded from the odds ratio (OR) calculations for shock because there were no in-hospital deaths at these sites in the shock cohort, resulting in a final traumatic shock study cohort of 582 patients. This included 103 patients (17.7%) with hypoxemia (PaO_2_ <80 mm Hg), 429 patients (73.7%) with normoxemia (PaO_2_ 80–400 mm Hg), and 50 patients (8.6%) with hyperoxemia (PaO_2_ >400 mm Hg). Baseline demographic and clinical data are displayed in Tables 1B and 2B. A total of 52 traumatic shock patients (9%) died before hospital discharge.

For the TBI cohort, multiple logistic regression analysis revealed statistically significant associations between hypoxemia and higher risk of in-hospital mortality (OR, 1.8; 95% CI, 1.2–2.8; *p* = 0.008) and between hyperoxemia and lower risk (OR, 0.6; 95% CI, 0.4–0.9; *p* = 0.018). For the traumatic shock cohort, multiple logistic regression analysis did not reveal a statistically significant association between either hypoxemia and mortality (OR, 1.0; 95% CI, 0.4–2.4; *p* = 0.986) or between hyperoxemia and mortality (OR, 1.9; 95% CI, 0.6–5.7; *p* = 0.269). These data are displayed in Tables 3A (TBI cohort) and 3B (traumatic shock cohort).

**Table 3A.** Logistic Regression Analysis for All TBI Patients

Oxygenation	Odds ratio	95% Confidence intervals	*p* value
Hypoxemia	1.8	(1.2, 2.8)	0.008
Hyperoxemia	0.6	(0.4, 0.9)	0.018

Odds ratios of death were adjusted for site, age, sex, mechanism of injury, GCS [TBI cohort], advanced airway insertion, hypo- or hypercapnia, ISS, pre-hospital SBP, Head AIS, Chest AIS, and Abdomen AIS.

TBI, traumatic brain injury; GCS, Glasgow Coma Scale; ISS, Injury Severity Score; SBP, systolic blood pressure; AIS, Abbreviated Injury Score.

**Table 3B.** Logistic Regression Analysis for All Traumatic Shock Patients

Oxygenation	Odds ratio	95% Confidence intervals	*p* value
Hypoxemia	1.9	(0.6, 5.7)	0.269
Hyperoxemia	1.0	(0.4, 2.4)	0.986

Odds ratios of death were adjusted for site, age, sex, mechanism of injury, GCS [TBI cohort], advanced airway insertion, hypo- or hypercapnia, ISS, pre-hospital SBP, Head AIS, Chest AIS, and Abdomen AIS.

GCS, Glasgow Coma Scale; TBI, traumatic brain injury; ISS, Injury Severity Score; SBP, systolic blood pressure; AIS, Abbreviated Injury Score.

**FIG. 2.**
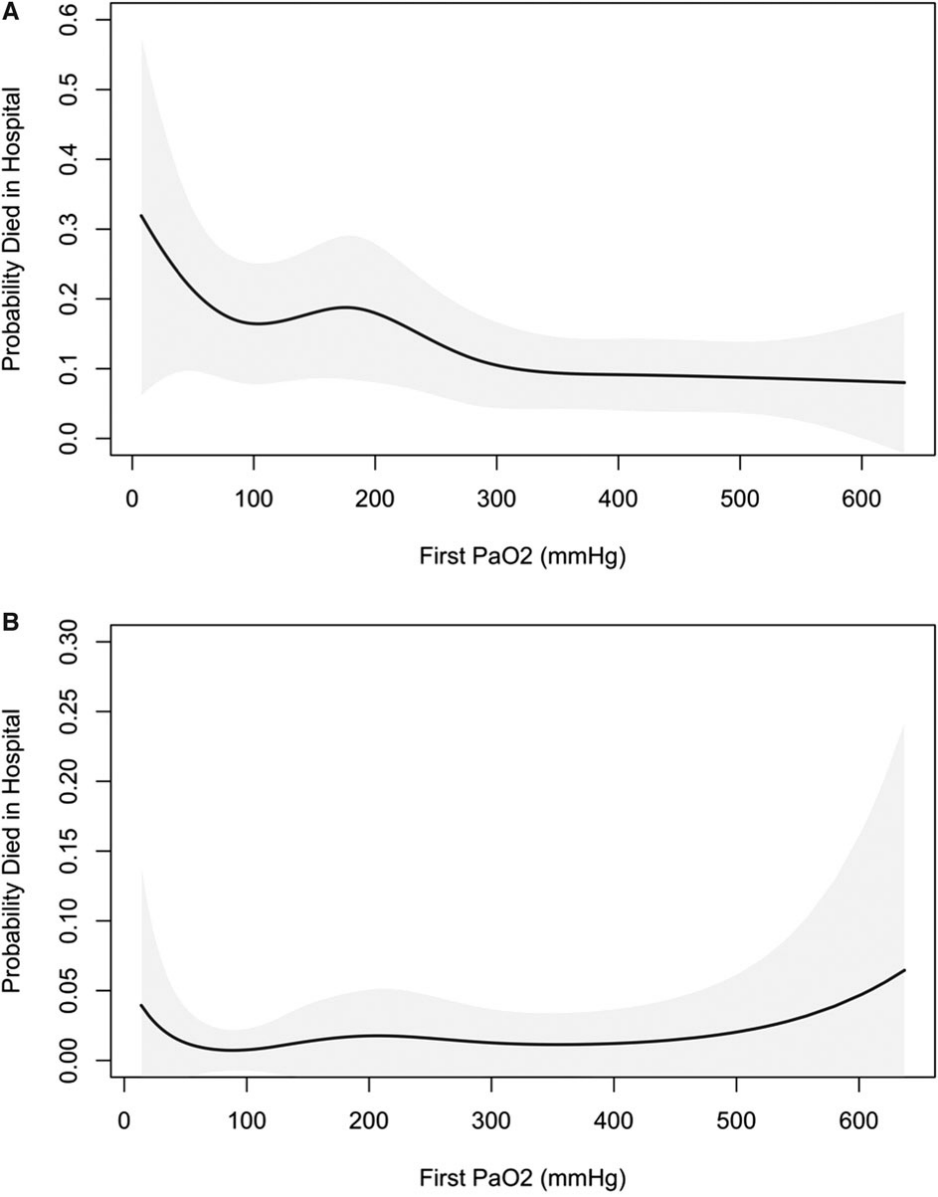
Spline curve for oxygenation and outcome for all TBI patients (**A**) and traumatic shock patients (**B**). (A) Spline curve and 95% point-wise CI relating oxygenation to probability of dying before hospital discharge among 40-year-old, eucapneic male TBI patients at one site who suffered blunt injury, were not administered an advanced airway, and who had an ISS of 26, lowest pre-hospital SBP of 110 mm Hg, maximal head AIS of 4, and maximal chest and abdomen AIS of 0. (B) Spline curve and 95% point-wise CI relating oxygenation to probability of dying before hospital discharge among 40-year-old, eucapneic male traumatic shock patients at one site who suffered blunt injury, were not administered an advanced airway, and who had pre-hospital GCS >8, ISS of 26, lowest pre-hospital SBP of 80 mm Hg, and maximal head, chest, and abdomen AIS of 0. AIS, Abbreviated Injury Scale; CI, confidence interval; GCS, Glasgow Coma Scale; ISS, Injury Severity Scale; SBP, systolic blood pressure; TBI, traumatic brain injury.

Regression spline plots for the association between initial PaO_2_ and the probability of death before hospital discharge in these two cohorts are displayed in Figure 2A,B. For TBI patients, mortality appeared to be highest with PaO_2_ values <100 mm Hg and lowest with PaO_2_ values >300 mm Hg. For traumatic shock patients, mortality appeared to be higher with extreme hypoxemia (PaO_2_ <60 mm Hg) or hyperoxemia (PaO_2_ >500 mm Hg), but the small number of patients in these subgroups resulted in wide CIs.

Deletion diagnostics were computed to examine the influence of individual observations on the OR estimates. There were no subjects the addition of whose data resulted in a ≥10% difference in the ORs reported in the TBI cohort. In the shock cohort, there were 10 subjects the addition of whose data resulted in a >10% difference on the OR associated with hyperoxemia, and 8 the addition of whose data resulted in a >10% difference on the OR associated with hypoxemia. When these 18 subjects were omitted from the shock cohort, OR estimates for the association between hyperoxemia and hypoxemia and in-hospital mortality were both <1, but CIs were wide and neither OR was statistically significantly different from 1, so that general conclusions in the shock cohort were not altered.

## Discussion

The physiological disruption that occurs after a serious injury may include hypoperfusion as well as hypoxemia, both of which may exacerbate primary brain injury, produce ischemic-hypoxic damage, or trigger an inflammatory cascade that can end in multi-organ failure and death. Thus, it is not surprising that optimal resuscitation of seriously injured patients has focused on rapidly reversing perfusion and oxygenation deficits through infusion of intravenous fluids, transfusion of blood, and both the non-invasive and invasive administration of supplemental oxygen.^6^ This rapid return to normal—and even supranormal—physiological values has represented the hallmark of successful resuscitative efforts despite the absence of clinical outcomes data to support this approach. Recently, concerns have been raised regarding an association between hyperoxemia and adverse outcomes in non-TBI, including birth asphyxia, shock, post-surgical patients, and cardiac arrest.^16–20^

Here, we use a large, multi-center registry database to explore the association between both hypoxemia and hyperoxemia and outcome in trauma patients with severe TBI and traumatic shock. Using thresholds determined *a priori*, we observed a relationship between hypoxemia and decreased survival for severe TBI, but not for traumatic shock. In addition, we observed an association between hyperoxemia and improved survival for severe TBI patients, which was counter to our hypothesis. Only the spline analysis for the subset of traumatic shock patients suggested an adverse effect of hyperoxemia on outcome. However, analyses were limited by the relatively small number of subjects in the traumatic shock cohort and even smaller number that died before hospital discharge. This study was notable for the large number of critically injured patients and the quality of data collected at a heterogeneous group of EMS systems.

The association between hypoxemia and adverse outcomes in TBI patients has been observed previously and provides the primary justification for early, aggressive airway management in the setting of traumatic coma.^31^ The magnitude of association presented here was larger than documented by other investigations. It is also notable that even normal systemic oxygenation values have been associated with cerebral hypoxia, possibly attributable to problems with cerebral oxygen diffusion in the setting of cerebral edema and blood–brain barrier (BBB) disruption.^32,33^ The spline analysis suggests a more conventional threshold for hypoxemia, consistent with the value pre-chosen for this analysis.

Our hypothesis that hyperoxemia may be associated with increased mortality was not supported by these data. Instead, PaO_2_ values >400 mm Hg were associated with improved survival in the TBI cohort. Although experimental data suggest that higher oxygen concentrations may exacerbate TBI, clinical studies have been inconsistent in demonstrating an association between hyperoxemia and worsened outcomes. Davis and colleagues used a modified Trauma and Injury Severity Score analysis in a single-site study of >3000 patients and observed increased mortality in patients with extreme hyperoxemia (PaO_2_ >500 mm Hg), regardless of intubation status.^21^ Similarly, Rincon and colleagues observed higher case-fatality rates among ventilated TBI patients with hyperoxia (PaO_2_ >300 mm Hg) as compared to normoxia (PaO_2_ 60–300 mm Hg).^24^ Brenner and colleagues also found worse outcomes among TBI patients with either hyperoxia or hypoxia, but limited their analysis to those surviving at least 12 h.^23^

Observations by other investigators support these findings of a potential harmful effect of excessive oxygen post-TBI.^34,35^ On the other hand, Taher and colleagues performed a randomized controlled trial to explore the impact of 50% versus 80% oxygen in TBI patients undergoing mechanical ventilation and observed improved long-term outcomes in the higher FiO_2_ cohort, which is more consistent with data presented here.^22^ Other investigators have also demonstrated potential metabolic benefits with early hyperoxemia.^36,37^ The spline plot in Figure 2A may offer an avenue for reconciling these apparently inconsistent observations. Once PaO_2_ values reached ∼300 mm Hg, little additional survival benefit was associated with higher oxygen levels. Similarly, Davis and colleagues observed peak survival rates with PaO_2_ values of 300–350 mm Hg.^21^ Thus, a reasonable therapeutic strategy would be to target PaO_2_ values ∼300–350 mm Hg, which would represent optimal survival rates across each of the above studies.

Although a complete explanation of these apparently disparate findings remains elusive, the complexity and heterogeneity of TBI almost certainly plays a role. Experimental models of brain injury clearly demonstrate both beneficial as well as potentially harmful effects of oxygen.^38^ Further, the natural course of TBI involves a dynamic interplay between vasomotor dysfunction causing both ischemia and hyperemia, intracranial hypertension, massive discoordinated depolarization involving glutamate-mediated excitotoxicity, and an inflammatory cascade that ultimately results in neuronal death attributable to necrosis and apoptosis.^39–41^ Blasiole and colleagues compared normoxemic (mean pO_2_ = 82 mm Hg) to moderately hyperoxemic (mean pO_2_ = 342 mm Hg) resuscitation in a murine model of TBI with hemorrhagic shock, demonstrating that the beneficial effects of oxygen supplementation appear to outweigh the potential adverse effects of an increase in oxygen tension.^42^ In addition, hyperbaric oxygen is emerging as a potential therapy for post-concussive syndrome.^43^ Thus, the net effect may depend on the pathophysiological conditions present—including the potential for traumatic shock—as well as the amount and timing of oxygen administration. Our use of ABG data within the first 30 min of hospital arrival may not be comparable to values obtained over the first hours or days of intensive care unit admission. In addition, variable injury patterns may result in a differential balance between the beneficial and harmful effects of oxygen in particular patient types. Further, defining hyperoxia is complex and may involve operational definitions (e.g., FiO_2_), systemic measures of hyperoxemia (e.g., PaO_2_), or actual brain tissue oxygen tension (e.g., PbrO_2_). The potential for acute pulmonary injury and acute respiratory distress syndrome as well as cerebral edema, ischemia, and disruption of the BBB may lead to gradients between FiO_2_, PaO_2_, and PbrO_2_. Finally, this study was observational, with the potential for confounding by variables we could not control influencing our conclusions.

Even less is known about the relationship between oxygenation and traumatic shock. A traditional understanding of the pathophysiology of hemorrhage suggests that acute blood loss results in a decrease in hemoglobin that would be exacerbated by concurrent hypoxemia and may be partially offset by an increase in PaO_2_. However, the small amount of dissolved oxygen in blood would also suggest that there is little to be gained with regard to tissue oxygen delivery by extreme hyperoxemia once existing hemoglobin is completely saturated. Although correction of hypoxemia with supplemental oxygen is standard therapy for patients in shock, supranormal oxygen targets may not improve outcomes and may be associated with increased mortality in critically ill patients with shock.^18,44,45^ The data presented here failed to demonstrate a statistically significant association between either hypoxemia or hyperoxemia in the traumatic shock cohort. Whereas the spline curve suggests an increase in mortality with extreme hypoxemia and hyperoxemia, the small number of patients and deaths in these categories resulted in wide CIs and an absence of statistically significant differences in mortality when compared to normal oxygenation.

The use of invasive airway devices in the pre-hospital environment has been associated with increased mortality among severely injured patients, particularly those with TBI.^11–13^ It is notable that an advanced airway was inserted in around half of the TBI cohort patients, but <10% of the traumatic shock cohort patients. Although this reflects a decreased level of consciousness—one of the definitions for TBI—as an indicator for aggressive airway management, this also may influence the balance between therapeutic and potentially harmful effects of oxygen in these patients. Further, the inverse relationship between PaO_2_ and PaCO_2_ is intriguing, given that hyperventilation and hypocapnia have repeatedly been associated with higher mortality.^14,15^ The perception of a therapeutic “trade-off” between oxygenation and ventilation has been described previously and warrants further exploration.

These observations must be considered in the context of the study limitations. Our definitions for hypoxemia and hyperoxemia were based on previous investigations, but may not represent brain tissue hypoxia or hyperoxia, which may not correlate perfectly with ABG measurements and likely vary with each individual patient. The spline analysis, which helps address the possibility of imprecise definitions for hypoxemia and hyperoxemia, did not suggest that alternative thresholds would have led to a different result. In addition, we selected a somewhat arbitrary 30-min cutoff for the inclusion of ABG data after hospital arrival. Though this was intended to reflect oxygen delivery during early resuscitation, it is possible that this value was too long or short to detect a true association between oxygenation status and outcome. It is also possible that the requirement for data from an ABG obtained within 30 min of arrival introduces a form of selection bias.

Further, the extreme pO_2_ values used to define hyperoxemia may introduce a form of selection bias that reflects the absence of severe lung injury. Although we attempted to adjust for this using Chest AIS scores, more precise measurements of lung function, such as the P/F ratio, were not available. However, we would expect Chest AIS scores to better reflect P/F ratio abnormalities early post-injury as compared to later in the hospitalization, when acute lung injury and acute respiratory distress syndrome typically emerge. Similarly, measures of perfusion, such as cardiac output, oxygen extraction ratios, or vasopressor requirements, and functional outcome measures were not included in the PROPHET database.

Detecting a potential association between oxygenation and outcome is almost certainly influenced by the heterogeneity of traumatic injury. Our definitions for severe TBI and shock represent traditional GCS and SBP thresholds, but certainly do not reflect the richness and complexity of traumatic injury and may not have accurately identified patients with true brain injury or life-threatening hemorrhage. This is perhaps best reflected by the observation that 20% of patients had Head AIS scores of ≤2. In addition, the impact of varying oxygenation levels may have been better elucidated by some functional measure of brain function rather than survival-to-hospital discharge. Further, we did not attempt to define the timing of death, and it is conceivable that early mortality represents a different pathophysiology than later deaths.^5^ Although ROC sites prioritized the standardization of data definitions and consistency of subject enrollment, the use of patients from multiple EMS systems and trauma centers introduces the potential for intersite heterogeneity masking true associations between variables of interest. Finally, the relatively small number of patients—and even smaller number of deaths—in the traumatic shock cohort, particularly those with either hypoxemia or hyperoxemia, as well as the potential for confounding variables, limits our ability to draw firm conclusions.

## Conclusions

Among TBI patients, hypoxemia was associated with worse outcomes whereas hyperoxemia was associated with improved outcomes as compared to normoxemia. In the traumatic shock cohort, the spline analysis suggested a potential association between extremes of oxygenation and decreased survival, but the relatively small number of subjects and few deaths in this cohort resulted in wide CIs and an absence of statistically significant findings on regression analysis. Additional investigation as to the timing of oxygen delivery, as well as the potentially differential balance between the beneficial and harmful effects of oxygen therapy in various patterns of TBI, is warranted.

## Abbreviations Used

ABG: arterial blood gas
AIS: Abbreviated Injury Scale
BBB: blood–brain barrier
CI: confidence interval
EMS: emergency medical services
GCS: Glasgow Coma Sclae
ISS: Injury Severity Score
OR: odds ratio
ROC: Resuscitation Outcomes Consortium
SBP: systolic blood pressure
TBI: traumatic brain injury
